# Anti-Apoptotic and Anti-Inflammatory Properties of Grapefruit IntegroPectin on Human Microglial HMC3 Cell Line

**DOI:** 10.3390/cells13040355

**Published:** 2024-02-18

**Authors:** Miriana Scordino, Giulia Urone, Monica Frinchi, Chiara Valenza, Angela Bonura, Chiara Cipollina, Rosaria Ciriminna, Francesco Meneguzzo, Mario Pagliaro, Giuseppa Mudò, Valentina Di Liberto

**Affiliations:** 1 Dipartimento di Biomedicina, Neuroscienze e Diagnostica Avanzata, Università di Palermo, Corso Tukory 129, 90134 Palermo, Italy; miriana.scordino@unipa.it (M.S.); giulia.urone@unipa.it (G.U.); monica.frinchi@unipa.it (M.F.); chiara.valenza@unipa.it (C.V.); giuseppa.mudo@unipa.it (G.M.); 2 Istituto di Farmacologia Traslazionale, CNR, Via U. La Malfa 153, 90146 Palermo, Italy; angela.bonura@ift.cnr.it; 3 Fondazione RiMED, Via Ugo La Malfa 153, 90146 Palermo, Italy; ccipollina@fondazionerimed.com; 4 Istituto per lo Studio dei Materiali Nanostrutturati, CNR, Via U. La Malfa 153, 90146 Palermo, Italy; rosaria.ciriminna@cnr.it (R.C.); mario.pagliaro@cnr.it (M.P.); 5 Istituto per la Bioeconomia, CNR, Via Madonna del Piano 10, Sesto Fiorentino, 50019 Florence, Italy; francesco.meneguzzo@cnr.it

**Keywords:** pectin, microglia, oxidative stress, neuroinflammation, Akt, ERK, NF-kB, iNOS

## Abstract

In this study, we investigated the beneficial effects of grapefruit IntegroPectin, derived from industrial waste grapefruit peels via hydrodynamic cavitation, on microglia cells exposed to oxidative stress conditions. Grapefruit IntegroPectin fully counteracted cell death and the apoptotic process induced by cell exposure to tert-butyl hydroperoxide (TBH), a powerful hydroperoxide. The protective effects of the grapefruit IntegroPectin were accompanied with a decrease in the amount of ROS, and were strictly dependent on the activation of the phosphoinositide 3-kinase (PI3K)/Akt cascade. Finally, IntegroPectin treatment inhibited the neuroinflammatory response and the basal microglia activation by down-regulating the PI3K- nuclear factor kappa-light-chain-enhancer of activated B cells (NF-kB)- inducible nitric oxide synthase (iNOS) cascade. These data strongly support further investigations aimed at exploring IntegroPectin’s therapeutic role in in vivo models of neurodegenerative disorders, characterized by a combination of chronic neurodegeneration, oxidative stress and neuroinflammation.

## 1. Introduction

Neurological disorders (NDs), including neurodegenerative diseases, are one of the most impactful conditions afflicting contemporary society wellness, being the second cause of death worldwide [1]. The growth of the global population, aging and unhealthy and industrialized lifestyles are leading to an exponential increase in the burden of NDs. Moreover, NDs emerge as multifactorial diseases, sharing a pathogenic pathway that includes mitochondrial dysfunction, oxidative stress, misfolded protein aggregation, and neuroinflammation. Consequently, despite decades of clinical and basic research, most therapeutic strategies designed to manage degenerative NDs are only palliative and often associated with a broad spectrum of side effects [2].

Microglia cells, descending from the myeloid lineage, are considered the resident immune cells of the brain. In a physiological context, resting microglia show a ramified body, which allows them to oversee the neuronal status and the biochemical balance of the surrounding area by producing neurotrophic factors, executing the synaptic pruning, and exerting phagocytic activity on cellular debris. Under pathological chronic triggers, microglia change morphology and activate a cascade of events which leads to the failure of the brain’s homeostasis surveillance, the activation of an inflammatory response, and enhances the neurodegeneration process [3]. Neuroinflammation and neurodegeneration are sustained by the release of pro-inflammatory cytokines, including interleukin (IL)-1β and IL-6, chemokines, small-molecule messengers such as prostaglandins and nitric oxide (NO), and the activation of molecular pathways involving the nuclear factor kappa-light-chain-enhancer of activated B cells (NF-κB) and reactive oxygen species (ROS) [4]. In particular, the abnormal amount of ROS and the establishment of oxidative stress create a synergy with the neuroinflammatory response, leading to neurodegeneration [5,6]. For all these reasons, microglia cells represent an important target for the development of therapeutic strategies, and the full understanding of their response under basal and pathological conditions is essential in the context of preclinical and clinical neuroscience research [7].

So far, synthetic drugs have failed in preventing or treating neurodegenerative diseases. Plentiful research efforts, thus, have been focused on the possible use of natural and bioactive compounds derived from plants, fruits, and seeds exerting antioxidant and anti-inflammatory actions [8,9,10]. A common neuroprotective mechanism for widely different natural compounds derives from their ability to scavenge the excess of the free radicals generated in oxidative and neurotoxin-induced processes in the brain’s nerve cells [11]. In the context of these studies, we have lately shown the powerful neuroprotective, antioxidant and mitoprotective activity of lemon [12] and grapefruit [13] IntegroPectin in neuronal-like cells. “IntegroPectin” is the name of a new family of *Citrus* pectins obtained via the hydrodynamic cavitation of *Citrus* fruit processing’s industrial biowaste that is rich in adsorbed flavonoids [14] and terpenes [15]. In the first in vivo experiments, grapefruit IntegroPectin has recently shown anti-ischemic cardioprotective activity, significantly higher than that of pure naringenin, namely the bioactive aglycone of naringin [16]. Indeed, the amount of naringin present in grapefruit IntegroPectin, is very high (73.66 mg/g) [14]. Furthermore, the uniquely high solubility of IntegroPectin compared to that of commercial citrus pectin largely enhances the bio-availability of the poorly water-soluble, yet highly hydroxylated, naringin molecule, with the IntegroPectin’s poorly esterified pectic molecules in solution acting as drug carriers [17].

In this study, we investigated the anti-apoptotic and anti-inflammatory properties of grapefruit IntegroPectin on the human microglial clone 3 (HMC3) cell line, focusing on the modulation of specific intracellular signalling cascades. In more detail, we explored the activation of both the mitogen-activated protein kinase (MAPK)/ERK and the phosphoinositide 3-kinase (PI3K)/Akt pathways, and their downstream mediators, widely considered to be key pleiotropic modulators of several cellular processes, including inflammation and survival [18]. HMC3 cells retain most of the morphological and phenotypical characteristics of the primary source: they look like globular or elongated cells with thick processes and dark cytoplasmic granulation; they are positive for microglial and monocyte markers such as the Cluster of Differentiation 68 (CD 68) and the ionized calcium-binding adapter molecule 1 (IBA1); and they are able to release pro-inflammatory factors, such as IL-6, even under a basal condition. HMC3 cells can be activated by different stimuli, including pro-oxidant and neuroinflammatory activators [7]. In the present study, HMC3 cells were challenged with tert-butyl hydroperoxide (TBH), a relatively stable hydroperoxide [19], in the presence of grapefruit IntegroPectin, and the protective response was measured.

## 2. Materials and Methods

### 2.1. Solubilization of IntegroPectin

Grapefruit IntegroPectin, obtained as previously described from the *Citrus paradisi* processing’s industrial waste [14] kindly donated by Campisi Citrus (Siracusa, Italy), was solubilized at the concentration of 10 mg/mL in a cell culture medium. The solution was filtered using a 0.45 µm filter (Sartorius) and stored at 4 °C.

### 2.2. Cell Cultures and Treatments

HMC3 cells, generously donated by Prof. M. Sortino (University of Catania), were cultured in T25 tissue culture flasks in a humidified atmosphere of 95% air and 5% CO_2_ at 37 °C. The growth medium was composed by MEM (Minimum Essential Medium)–w/Earle’s Salts, supplemented with a 10% fetal bovine serum (FBS), 100 U/mL of penicillin, 100 U/mL of streptomycin, 2 mM of l-glutamine and non-essential amino acids. The cell culture medium was changed once a week and the cells were sub-cultured once they reached a 90% confluence. All treatments were performed at least 48 h after plating. Based on the experimental groups, the cells received the following treatments: IntegroPectin 1 mg/mL, 0.1 mg/mL and 0.01 mg/mL for 24 h in concentration-effect experiments; IntegroPectin 1 mg/mL for 24 h, 48 h and 72 h in time-course experiments; TBH 500 µM, 300 µM, 200 µM, 100 µM and 50 µM for 24 h in concentration-response experiments; a combination of IntegroPectin (1 mg/mL) and TBH (200 µM), with IntegroPectin administered immediately before (co-treatment) TBH treatment (24 h); in experiments requiring treatment with PD98059 (1213 Tocris Biotechne, Milano, Italy), an inhibitor of MAPK, and LY294002 (440202 Sigma-Aldrich Merck KGaA, Darmstadt, Germany), a PI3K/Akt inhibitor [20], the compounds (30 µM and 10 µM, respectively) were administered 1 h before IntegroPectin and TBH exposure. The concentrations of the two inhibitors were selected based on manufacturer instructions and the broad existing literature [21,22]. For experiments aimed at assessing the modulation of mRNA expression and acute intracellular pathway modulation, cells were treated for 4 h with IntegroPectin 1 mg/mL. In all experiments, the control group was treated with an equal volume of the cell medium.

### 2.3. Cell Viability by MTT Assay

Cells were grown at a density of 2.5 × 10^4^ cells/well in 96-well plates in a final volume of 100 µL/well. The cellular metabolic activity, an indicator of cell viability, was assessed by measuring the intracellular reduction of tetrazolium salt (MTT, 0.5 mg/mL) to purple formazan granules by the mitochondrial succinate dehydrogenase expressed in metabolically active cells after 3 h of incubation at 37 °C. Absorbance was measured at 570 nm with background subtraction after solubilizing an MTT–formazan product with dimethyl sulfoxide (DMSO), 100 µL/well. Cell viability was expressed as a percentage with the control group set to 100.

### 2.4. Nuclear Morphology by 4′,6-diamidino-2-phenylindole (DAPI) Staining

For the analysis of cell nuclei morphology, cells were grown at a density of 2 × 10^5^ cells/well on coverslips placed in a 24-well plate in a final volume of 400 µL/well. Cells were fixed with a 4% formaldehyde solution for 15 min at room temperature and, after two washings with a phosphate-buffered saline (PBS), nuclei were counterstained with the fluorescent stain (DAPI). After one washing with PBS, the coverslips were mounted on slides, and the cellular images were obtained using a fluorescence microscope (DMRBE, Leica Microsystems GmbH, Wetzlar, Germany), equipped with a digital video camera (Spot-RT Slider, Diagnostic Instruments, Sterling Heights, MI, USA).

### 2.5. Quantification of Reactive Oxygen Species (ROS) via Dichlorofluorescein Diacetate (DCFH-DA) Assay

HMC3 cells were placed at a density of 2.5 × 10^4^ cells/well in 96-well plates in a final volume of 100 µL/well. After exposure to the indicated treatments, cells were incubated in the dark for 10 min at room temperature with DCFH-DA (10 µM), washed with PBS and the fluorescence intensity was measured using the microplate reader GloMax fluorimeter (Promega Corporation, Madison, WI, USA) at the excitation wavelength of 475 nm and emission wavelength 555 nm. Results were expressed as percentages with the control group set at 100.

### 2.6. Western Blotting Analysis

To assess the Caspase-3, phosphorylated (p)-Extracellular signal-regulated kinase (ERK)1/2, p-Akt and p-NF-kB protein expression levels, HMC3 cells were placed at a density of 2.5 × 10^4^ cells/well in 96-wells plates in a final volume of 100 µL/well. At the end of treatment, for each experimental condition, cells were mechanically detached and the cell content of 4 wells was pooled into one sample. The cell pellet was homogenized in a cold radioimmunoprecipitation assay (RIPA) buffer (50 mM Tris, pH 7.4, 150 mM NaCl, 1% Triton, SDS 0.1%), supplemented with a protease inhibitor cocktail (Sigma-Aldrich P8340) and a phosphatase inhibitor cocktail (P5726 Sigma-Aldrich Merck KGaA, Darmstadt, Germany) and sonicated (30 pulsations/min). Proteins were quantified via the Lowry method [23]. A western blotting procedure was performed as previously described [24]. The protein samples (30 µg per lane) and the molecular weight marker (PageRuler Plus Prestained Protein Ladder, 26619 ThermoFisher Scientific, Waltham, MA USA) were run on an 8% polyacrylamide gel and electrophoretically transferred onto a nitrocellulose membrane (RPN303E, Hybond-C-extra, GE Healthcare Europe, Milano, Italy). The membranes were incubated at 4 °C under gentle shaking for 1 h in a blocking buffer (1× TBS, 0.1% Tween−20, 5% *w*/*v* nonfat dry milk), followed by overnight incubation with specific antibodies diluted in a blocking buffer. For the detection of Caspase-3, p-ERK1/2, p-Akt, and p-NF-kB levels, the following antibodies were used: mouse anti-Caspase-3 (1:400, sc-56053 Santa Cruz Biotechnology, Dallas, TX, USA), rabbit anti-p-ERK1/2 (1:1000, 9101 Cell Signaling Technology, Beverly, MA, USA), rabbit anti-p-Akt (1:1000, 4060 Cell Signaling Technology, Beverly, MA, USA), and mouse p-NF-kB (1:500, sc-136548 Santa Cruz Biotechnology, Dallas, TX, USA). The day after, membranes were washed three times for 10 min with TBS/T and incubated for 1 h with goat anti-mouse IgG-HRP (sc-2005 Santa Cruz Biotechnology, Dallas, TX, USA) or mouse anti-rabbit IgG-HRP (sc-2357 Santa Cruz Biotechnology, Dallas, TX, USA) horseradish peroxidase conjugated diluted at a ratio of 1:10,000. After three washings with the TBS-T, the immunocomplexes were visualized with a chemiluminescence reagent (SuperSignal West Pico Plus, ThermoFisher Scientific, Waltham, MA, USA). The membranes were exposed to an autoradiography film (28-9068-36 Amersham Hyperfylm ECL, GE Healthcare Europe, Milano, Italy). The chemiluminescent signal was visualized and fixed in a Kodak D19 developer and fixer (1900984 and 1902485, Kodak GBX, Rochester, NY, USA). A sample of chemiluminescent membranes was developed using the iBright FL1000 Imaging System (ThermoFisher Scientific, Waltham, MA, USA). For western blotting normalization, membranes were washed and exposed to a horseradish peroxidase-conjugated β-Actin primary antibody (sc-47778 Santa Cruz Biotechnology, Dallas, TX, USA), diluted at a ratio of 1:10,000, for 1 h. The densitometric evaluation of bands was performed by measuring the optical density (O.D.) using ImageJ software (v. 1.53k, Rasband, W.S., ImageJ, U. S. National Institutes of Health, Bethesda, MD, USA, https://imagej.net/ij/ (accessed on 1 January 2024), 1997–2018) with results being expressed as arbitrary units. 

### 2.7. Cytofluorimetry Analysis for the Annexin V/Propidium Iodide (PI) Apoptosis Assay

HMC3 cells were plated at a density of 2.5 × 10^4^ cells/well in 96-well plates in a final volume of 100 µL/well. At the end of treatment, cells were mechanically detached and centrifuged at 1200 rpm for 5 min at room temperature. After discarding the supernatant, samples were resuspended in cold PBS 1X −/− (no calcium, no magnesium) (Gibco, Thermo Fisher Scientific Waltham, MA USA) and centrifuged. Cells were resuspended at 1 × 10^5^ cells/ in 100 μL of Annexin-binding buffer 1X (Invitrogen, Thermo Fisher Scientific, Waltham, MA, USA) and 5 µL of Alexa Fluor 647 Annexin V (Invitrogen, Thermo Fisher Scientific) diluted at a ratio of 1:10 with Annexin V binding buffer 1X were added to each 100 µL of cell suspension. An unstained control was prepared. All the samples were incubated at room temperature for 15 min in the dark. After incubation, cold PBS was added and cells were centrifuged, the supernatants discarded to remove any excess of unbound Annexin V, resuspended in 200 µL of Annexin-binding buffer 1X and kept on ice. PI (1 µL/100 µL) was added 5 min before starting the reading on the flow cytometer CytoFlex S (Beckman Coulter, Brea, CA, USA). Results were shown and analyzed through CytExpert flow cytometry analysis software. For data analysis, the gating strategy applied is based on the exclusion of doublets by the forward scatter physical parameter for the area (FSC-A) and for the height (FSC-H). The plots for Annexin V vs. PI are gated on the resulting populations. Details are described in the Results.

### 2.8. RNA Isolation and Reverse Transcription PCR

HMC3 cells were cultured at a density of 2.5 × 10^4^ cells/well in 96-wells plates in a final volume of 100 µL/well. At the end of treatment, for each experimental condition, cells were mechanically detached and the cell content of 7 wells was combined into one sample. The RNA was isolated from the cell pellet using a Qiagen RNeasy mini kit (74104, Qiagen, Hilden, Germany) following the manufacturer’s instructions. The total RNA concentration was detected using a MultiskanGO Microplate Spectrophotometer (Thermo Scientific Waltham, MA USA) and 1 μg was immediately retro-transcribed using a mixture containing: a 5× first strand buffer (18080-044, Invitrogen, Thermo Fisher Scientific, Waltham, MA, USA), 2.5 μM of random hexamers (N112470, Roche, Basel, Switzerland), 2.5 mM of dithiothreitol (DTT) (18080-044, Invitrogen, Thermo Fisher Scientific, Waltham, MA, USA), 0.5 mM of dNTPs mix (18427-013, Invitrogen, Thermo Fisher Scientific, Waltham, MA, USA), 40U of RNAse inhibitor (N8080119, Invitrogen, Thermo Fisher Scientific, Waltham, MA, USA), and 200 U of Superscript III Reverse Transcriptase (18080-044, Invitrogen, Thermo Fisher Scientific, Waltham, MA, USA). Reaction mixtures (20 μL) were incubated for 2 h at 50 °C and then for 15 min at 70 °C.

### 2.9. Real-Time PCR

A quantitative gene expression analysis of IL-6, IL-1β, and inducible nitric oxide synthase (iNOS) was performed using a SYBR Green Real-time PCR. The reaction was carried out in a total volume of 20 μL containing: a Power Track SYBR Green Master mix (A46109, AppliedBiosystem, Thermo Fisher Scientific, Waltham, MA, USA), 2 μL of cDNA, and 0.6 μM of primers mix (Forward (5′ to 3′): IL-6 CACTGGTCTTTTGGAGTTTGAG; IL-1β GCCAGTGAAATGATGGCTTATT; iNOS GACTTTCCAAGACACACTTCAC; Reverse (5′ to 3′) IL-6 GGACTTTTGTACTCATCTGCAC; IL-1β AGGAGCACTTCATCTGTTTAGG; iNOS TTCGATAGCTTGAGGTAGAAGC). The real-time PCR was performed in 48-well plates using the Step-One Real-Time PCR System (Applied Biosystems). Relative changes in the gene expression between control and treated samples were determined using the 2 (-Delta Delta CT) method. Levels of the target transcript were normalized to β-actin levels (Forward primer: TCCCTTGCCATCCTAAAAGCCACCC; Reverse primer: CTGGGCCATTCTCCTTAGAGAGAAG). Final values were expressed as fold changes.

### 2.10. Statistical Analysis

Data analysis was performed using GraphPad Prism 9.0.2 software (GraphPad Software, La Jolla, CA, USA). The normal distribution of data was assessed via the Shapiro–Wilk test. Statistical evaluations were performed using a one-way ANOVA, followed by Tukey Post hoc test. A *t*-test was used for the statistical comparison between the means of two groups. The relative results were presented as the mean ± SE of at least three independent experiments. Differences in *p*-value less than 0.05 were considered statistically significant.

## 3. Results

### 3.1. Effects of Grapefruit IntegroPectin on HMC3 Cell Viability

**Figure 1:**
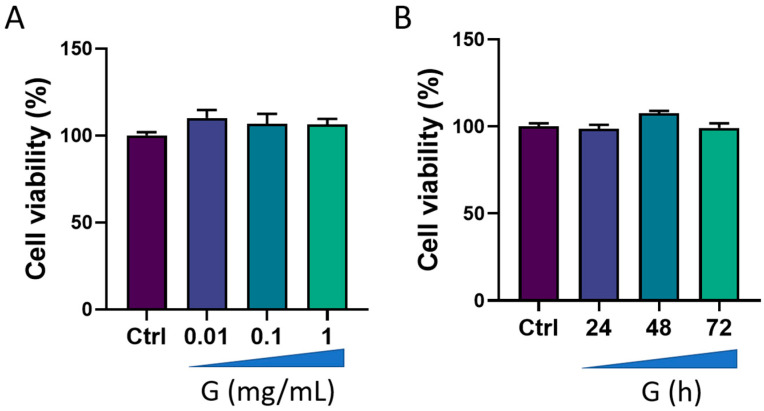
Effects of grapefruit IntegroPectin (G) on HMC3 cell viability. (**A**) Concentration–effect of G treatment (24 h) on cell viability, evaluated via MTT assay. (**B**) Time-course of G treatment (1 mg/mL) effects on cell viability, assessed via MTT assay.

We first evaluated the viability of HMC3 cells after treatment with grapefruit IntegroPectin. Results from concentration-response experiments indicated no changes in cell viability following cell exposure for 24 h to three different concentrations of IntegroPectin: 0.01 mg/mL, 0.1 mg/mL and 1 mg/mL (Figure 1A). Next, we carried out a time-course experiment by treating cells with the highest concentration of IntegroPectin (1 mg/mL). Results in Figure 1B show that IntegroPectin did not induce any significant changes in cell viability, even when the treatment is prolonged up to 72 h. Based on these data, IntegroPectin 1 mg/mL was used in all the subsequent experiments. 

### 3.2. Protective and Antioxidant Effects of Grapefruit IntegroPectin in HMC3 Cells Treated with TBH

**Figure 2:**
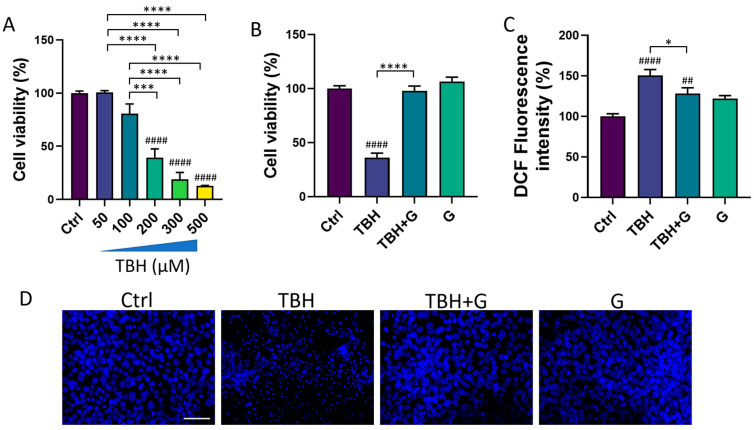
Protective and antioxidant effects of grapefruit IntegroPectin (G). (**A**) Concentration–response effects of TBH treatment (24 h) on HMC3 cell viability, evaluated via MTT assay. (**B**) Quantification of cell viability via MTT test in untreated (Ctrl) cells, cells treated with TBH (200 µM, 24 h), TBH (200 µM, 24 h) + G (1 mg/mL, 24 h), and G alone (1 mg/mL, 24 h). (**C**) DCF fluorescence intensity quantification, an index of intracellular ROS generation, in Ctrl cells, cells treated with TBH (200 µM, 24 h), TBH (200 µM, 24 h) + G (1 mg/mL, 24 h), and G alone (1 mg/mL, 24 h). (**D**) Representative pictures of DAPI nuclear staining in Ctrl cells, cells treated with TBH (200 µM, 24 h), TBH (200 µM, 24 h) + G (1 mg/mL, 24 h), and G alone (1 mg/mL, 24 h). Tukey test: ## *p* < 0.01, #### *p* < 0.0001 as compared with Ctrl group. * *p* < 0.05, *** *p* < 0.001, **** *p* < 0.0001. Scale bar: 100 μm.

We then tested the ability of IntegroPectin to recover the cell viability impaired by the oxidizing agent (TBH) treatment. HMC3 exposure for 24 h to TBH led to a concentration-dependent decrease in cell viability (Figure 2A). TBH 200 µM, which is able to induce a significant decrease in cell viability, was selected for all the subsequent experiments. To evaluate the protective effect of IntegroPectin, HMC3 cells were treated with IntegroPectin (1 mg/mL) immediately before TBH exposure. As shown in Figure 2B, IntegroPectin treatment fully prevented the cell death induced by the TBH treatment.

The impact of IntegroPectin on TBH-induced oxidative stress and ROS generation was assessed via a DCFH-DA fluorescence intensity assay. The fluorescence intensity measurement, an index of intracellular ROS levels, showed that the treatment of HMC3 cells with the grapefruit IntegroPectin significantly reduced intracellular ROS generation, boosted by TBH exposure (Figure 2C).

Moreover, the protective effects exerted by IntegroPectin treatment on cell viability following TBH exposure were further confirmed by qualitatively comparing the morphology of DAPI-stained cell nuclei. The bright fluorescent nuclei of TBH-treated cells appear smaller, fragmented and condensed, as compared to the control nuclei, while treatment with grapefruit IntegroPectin prevented this morphological change (Figure 2D).

### 3.3. Anti-Apoptotic Effects of Grapefruit IntegroPectin in HMC3 Cells Treated with TBH

**Figure 3:**
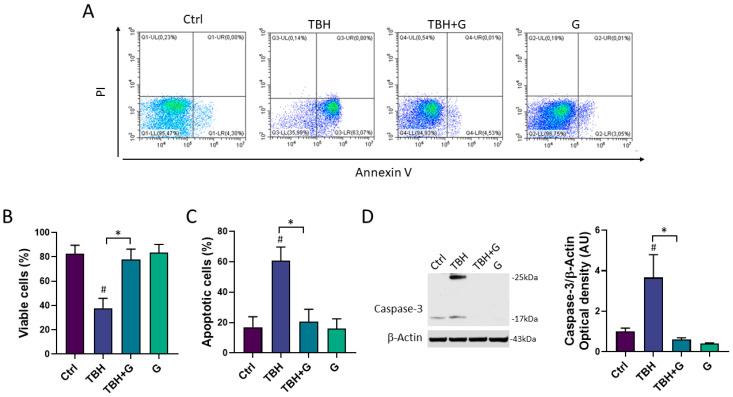
Anti-apoptotic effects of grapefruit IntegroPectin. (**A**) Representative plot indicating the percentage of Annexin V^-^ PI^-^ cells (on the lower left quadrant), Annexin V^+^ PI^-^ cells (on the lower right quadrant), PI^+^ Annexin V^-^ cells (on the upper left quadrant) and PI^+^ Annexin V^+^ cells (on the upper right quadrant) of different conditions: untreated sample (Ctrl), sample treated with TBH (200 µM, 24 h), sample co-treated with TBH (200 µM, 24 h) and IntegroPectin (G) (1 mg/mL, 24 h), and sample treated with IntegroPectin (G) alone (1 mg/mL, 24 h). (**B**) Histogram showing the cumulative Annexin V^-^ PI^-^ cell (viable cells) percentages from all the experiments. (**C**) Histogram showing the cumulative Annexin V^+^ PI^-^ cell percentages (apoptotic cells) from all the experiments. (**D**) Representative images of Caspase-3 and β-actin western blotting bands and histogram of Caspase-3 normalized to β-actin Optical density in Ctrl cells, cells treated with TBH (200 µM, 24 h), TBH (200 µM, 24 h) + G (1 mg/mL, 24 h), and G alone (1 mg/mL, 24 h). Tukey test: # *p* < 0.05 as compared to Ctrl group; * *p* < 0.05. AU (Arbitrary Units).

The cell death induced by TBH exposure is mainly mediated by the activation of the apoptotic process [19]. In order to study the ability of grapefruit IntegroPectin to inhibit the apoptosis induced by TBH treatment, cellular apoptosis/necrosis was evaluated via the flow cytometry quantification of the Annexin V binding and PI uptake. In Figure 3A, values within the representative density plots indicate the percentage of viable Annexin V^−^ PI^−^ cells, intermediate apoptotic Annexin V^+^ PI^−^ cells, necrotic Annexin V^−^ PI^+^ cells, and late apoptotic Annexin V^+^ PI^+^ cells. Plots show that cells in the untreated (Ctrl) sample retained high viability (Annexin V^−^ PI^−^, 95.5%) with only a small percentage of Annexin V^+^ PI^−^ cells (4.3%) undergoing intermediate apoptosis. The oxidizing action of TBH affected the viability of the sample (Annexin V^−^ PI^−^, 36%) leading to a higher percentage of Annexin V^+^ PI^−^ cells (63%) indicative of intermediate apoptosis. In a co-treatment with TBH, IntegroPectin exerted a protective action as the sample yielded a high percentage of Annexin V^−^ PI^−^ cells (95%) and a small percentage (4.5%) of Annexin V^+^ PI^−^ cells, thus preventing oxidative stress-induced apoptosis. Treatment with IntegroPectin alone did not induce any change in cell viability and in the percentage of Annexin V^−^ PI^−^ cells (96.7%) and Annexin V^+^PI^−^ cells (3%), as compared to the Ctrl group. No significant percentages of necrotic cells (Annexin V^−^ PI^+^) or secondary necrotic cells (Annexin V^+^ PI^+^) show up in both density plots. Histograms in Figure 3B,C gather the cumulative percentages of the viable Annexin V^−^ PI^−^ cells and intermediate apoptotic Annexin V^+^ PI^−^ cells in the different experiments.

In order to further characterize the anti-apoptotic role of grapefruit IntegroPectin, the protein levels of Caspase-3, a thiol protease that acts as a major effector caspase involved in the execution phase of apoptosis [25], were explored. Treatment with the oxidizing agent alone (TBH 200 µM) induced a significant increase in the cleaved forms of Caspase-3 (25 kDa and 17 kDa), while grapefruit IntegroPectin was able to counteract this effect (Figure 3D).

### 3.4. Modulation of the MAPK/ERK and PI3K/Akt Pathways by Grapefruit IntegroPectin

**Figure 4:**
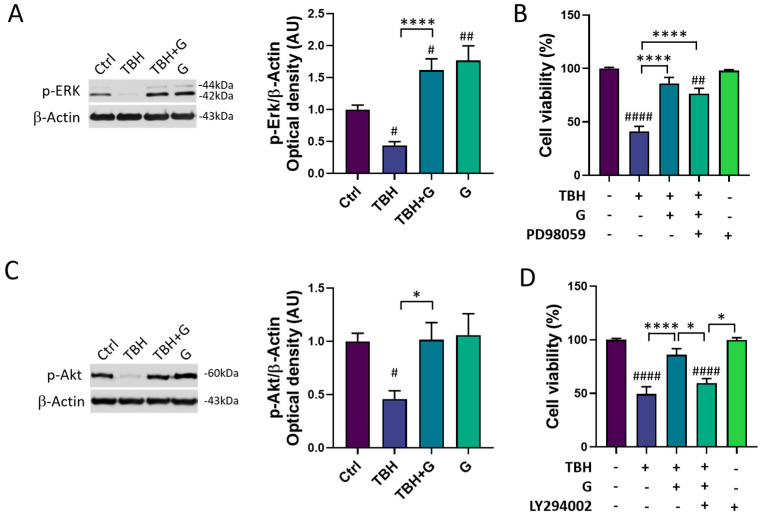
Modulation of MAPK/ERK and PI3K/Akt pathways by grapefruit IntegroPectin. (**A**) Representative images of phosphorylated (p)-ERK1/2 and β-Actin western blotting bands and histogram of p-ERK1/2 normalized to β-Actin Optical density in untreated (Ctrl) cells, cells treated with TBH (200 µM, 24 h), TBH (200 µM, 24 h) + IntegroPectin (G) (1 mg/mL, 24 h), and G alone (1 mg/mL, 24 h). (**B**) Quantification of cell viability via MTT test in Ctrl cells, cells treated with TBH (200 µM, 24 h), TBH (200 µM, 24 h) + G (1 mg/mL, 24 h), TBH (200 µM, 24 h) + G (1 mg/mL, 24 h) + PD98059 (30 µM), and PD98059 (30 µM) only. PD98059 was administered 1 h before grapefruit IntegroPectin and TBH exposure. (**C**) Representative images of phosphorylated (p)-Akt and β-Actin western blotting bands and histogram of p-Akt normalized to β-Actin Optical density in Ctrl cells, cells treated with TBH (200 µM, 24 h), TBH (200 µM, 24 h) + G (1 mg/mL, 24 h), and G (1 mg/mL, 24 h) alone. (**D**) Quantification of cell viability via MTT test in Ctrl cells, cells treated with TBH (200 µM, 24 h), TBH (200 µM, 24 h) + G (1 mg/mL, 24 h 10), TBH (200 µM, 24 h) + G (1 mg/mL, 24 h) + LY294002 (10 µM), and LY294002 (10 µM) only. LY294002 was administered 1 h before grapefruit IntegroPectin and TBH exposure. Tukey test: # *p* < 0.05, ## *p* < 0.01, #### *p* < 0.0001 as compared to Ctrl group; * *p* < 0.05, **** *p* < 0.0001. AU (Arbitrary Units).

In order to characterize the molecular mechanisms underlying grapefruit IntegroPectin’s protective response, we explored the modulation of two major cell survival pathways: MAPK/ERK and PI3K/Akt. As shown in Figure 4A, HMC3 cells treated with TBH (200 µM) show a significant down-regulation of phosphorylated (p)-ERK1/2 levels, which was fully counteracted by grapefruit IntegroPectin (G, 1 mg/mL) co-treatment. Surprisingly, grapefruit IntegroPectin alone was able to significantly enhance p-ERK protein levels, as compared to untreated (Ctrl) cells. In order to explore the specific involvement of the MAPK/ERK pathway in IntegroPectin’s protective function, HMC3 cells received a treatment with PD98059 (30 µM), a selective ERK1/2 signaling inhibitor, 1 h before grapefruit IntegroPectin (1 mg/mL) and TBH (200 µM) exposure (24 h). The treatment with PD98059 slightly reduced IntegroPectin’s protective effects against oxidative stress-induced cell death (Figure 4B), suggesting a minor involvement of MAPK/ERK signaling.

In addition to the p-ERK1/2 levels, IntegroPectin (1 mg/mL) co-treatment was able to fully recover the phosphorylated (p)-Akt protein levels, down-regulated by TBH exposure (200 µM, 24 h) (Figure 4C). Interestingly, when HMC3 cells were treated with LY294002 (10 µM), a selective PI3K/Akt inhibitor, 1 h before exposure to grapefruit IntegroPectin (G, 1 mg/mL) and TBH (200 µM, 24 h), the IntegroPectin’s protective activity completely vanished (Figure 4D), suggesting the major involvement of PI3K/Akt signaling in this effect. 

### 3.5. Modulation of Neuroinflammatory Pathways by Grapefruit IntegroPectin

**Figure 5:**
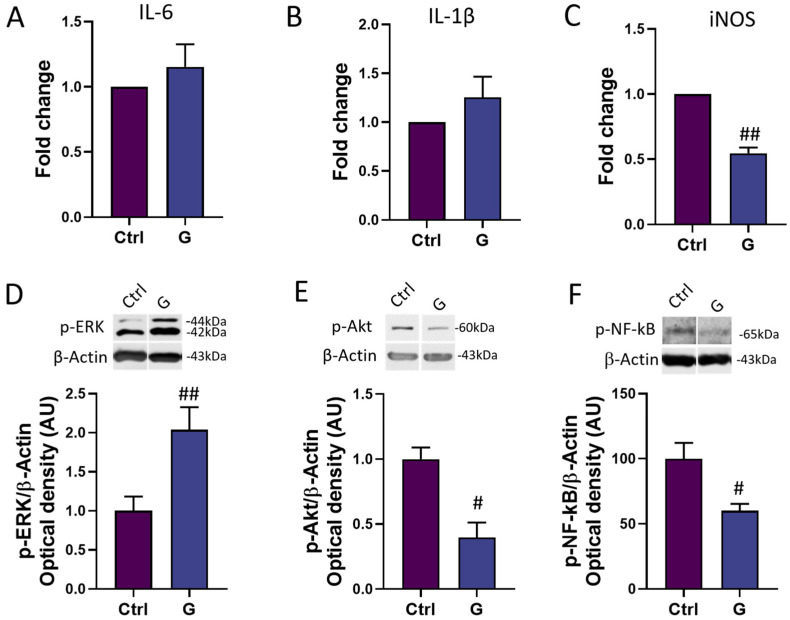
Modulation of inflammatory mediators and pathways by grapefruit IntegroPectin. Real-time PCR of IL-6 (**A**), IL-1β (**B**) and iNOS (**C**) mRNA levels in untreated (Ctrl) cells and cells treated with IntegroPectin (G) (1 mg/mL, 4 h). Representative images of phosphorylated (p)-ERK1/2 (**D**), p-Akt (**E**), p-NF-kB (**F**) and β-Actin western blotting bands and histogram of p-ERK1/2 (**D**), p-Akt (**E**) and p-NF-kB (**F**) normalized to β-Actin Optical density in Ctrl cells and cells treated with IntegroPectin (G) (1 mg/mL, 4 h). *t*-test: # *p* < 0.05, ## *p* < 0.01, as compared to Ctrl group. AU (Arbitrary Units).

In order to assess the potential anti-inflammatory power of grapefruit IntegroPectin, we investigated the mRNA expression of three major genes involved in the modulation of the inflammatory response in microglia cells: the main pro-inflammatory cytokines IL-6 and IL-1β, and the major downstream mediator of inflammation iNOS [26]. While IntegroPectin (G) treatment did not modulate the expression of IL-6 (Figure 5A) and IL-1β (Figure 5B), the bioactive molecule was able to significantly reduce the expression of iNOS, as compared to untreated (Ctrl) cells (Figure 5C). 

We also explored the time-related activation of three important intracellular modulators of the neuroinflammatory response: ERK1/2, Akt and NF-kB. Interestingly, a short-time (4 h) of IntegroPectin (G) treatment leads to a significant increase in p-ERK1/2 (Figure 5D) levels and a parallel decrease in p-Akt (Figure 5E) and p-NF-kB (Figure 5F) levels, as compared to Ctrl cells.

## 4. Discussion

Grapefruit IntegroPectin is a highly water-soluble *Citrus* pectin characterized by the preservation of the Rhamnogalacturonan-I (RG-I) backbone and endowed with large amounts of bioactive molecules, including flavonoids [14] and terpenes [15] well known for their robust anti-oxidant and anti-inflammatory properties. In line with the results of the present study, previous data have described the neuroprotective [13] effects of grapefruit IntegroPectin in neuronal-like cells and its anti-ischemic cardioprotective [16] activity, suggesting the activation of multi-target beneficial effects by this biopolymer. 

So far, the effects of IntegroPectin on microglia cells and the modulation of the neuroinflammatory response were unexplored. Through a combination of several experimental approaches, including nuclei morphology, Annexin V binding-PI uptake, and quantification of Caspase-3 levels, a protein crucial for apoptotic chromatin condensation and DNA fragmentation [27], we observed that grapefruit IntegroPectin is able to counteract TBH-induced apoptosis in the human microglial HMC3 cell line. We can speculate that the scavenger activity of grapefruit IntegroPectin against ROS blocks the cross-linked extrinsic and intrinsic apoptotic pathways, in the end re-establishing the mitochondrial homeostasis, as recently discovered in SH-SY5Y cells using both grapefruit [13] and lemon [12] IntegroPectin. Interestingly, while the grapefruit IntegroPectin treatment in SH-SY5Y cells produced a decrease in the proliferation rate and a cell cycle arrest at the G2/M phase, the same treatment did not affect cell viability and cell proliferation in HMC3 cells, suggesting the activation by IntegroPectin of cell type-specific effects.

Microglia exposure to TBH leads to a decrease in PI3K/Akt and MAPK/ERK signalling activation and both effects are counteracted by IntegroPectin treatment. PI3K/Akt and MAPK/ERK pathways have been widely described as pro-survival pathways [28], essential for cell protection against a plethora of noxious stimuli, including oxidative stress. Flavanones, for example, exert their neuroprotective activity by modulating the apoptosis process through the Akt and ERK1/2 signaling pathways [29]. Moreover, these two intracellular pathways are also involved in the neuroprotective effects activated by ginseng pectin treatment in cortical neuron cells [30]. Interestingly, using specific inhibitors, here we disclose a prominent role of the PI3K/Akt cascade in guaranteeing cell survival following cell exposure to TBH. 

More in general, both the MAPK/ERK and PI3K/Akt pathways represent a point of convergence of several cellular processes, including inflammation. Experimental evidence indicates that the PI3K/Akt pathway is required for the lipopolysaccharide (LPS) activation of microglial cells [31], while other studies suggest that PI3K/Akt activation contributes to the anti-inflammatory effects exerted by several biomolecules [32], thus outlining a complex scenario of both the protective and harmful responses orchestrated by this intracellular cascade. Similar results were obtained exploring the role of the MAPK/ERK pathway in inflammation modulation: an increase in ERK activation has been associated with the suppression of inflammatory genes in endothelial cells [33], and with the neuroinflammatory effects of Astragaloside IV in LPS-induced microglial cells [34]. Conversely, ERK is a critical regulator of the interferon (IFN) γ-mediated pro-inflammatory activation of microglia [35], and several anti-inflammatory drugs act through MAPK/ERK down-regulation [36]. These pieces of evidence suggest that several experimental variables, including the cell model, duration and typology of stimuli, may contribute to the state of activation of these pathways in the context of microglia physiology. 

In our cell model, a short (4 h) treatment with IntegroPectin is able to induce the up-regulation of MAPK/ERK activation, together with a down-regulation of the PI3K/Akt cascade. Interestingly, we observed that the IntegroPectin treatment of microglia cells leads also to a decrease in NF-kB activation, a transcriptional master regulator of the inflammatory and the apoptotic response [37]. The canonical NF-κB is activated in response to several external stimuli involved in inflammation, immune response, cell proliferation, differentiation, and survival, through the phosphorylation and subsequent degradation of the inhibitory IκB protein. On the other hand, the non-canonical NF-κB is selectively activated by TNF superfamily receptors which lead to the activation of NF-κB-inducing kinase (NIK), NIK-mediated p100 phosphorylation and the nuclear translocation of NF-κB [38]. We can hypothesize that the basal activation of the NF-κB pathway in HMC3 cells is likely dependent on the PI3K-Akt cascade, which regulates the transcriptional activity of NF-κB by inducing the phosphorylation and the subsequent degradation of the inhibitor of κB (IκB) [39]. By modulating Akt activation, grapefruit IntegroPectin leads to the subsequent down-regulation of NF-kB activity and the inflammatory response. In line with our results, recent studies described the beneficial activity of a modified citrus pectin in the inhibition of the NLRP3 inflammasome and NF-kB pathway activation in microglia during cerebral ischemia [40]. 

We also investigated the expression of a panel of genes typically associated with the pro-inflammatory response and the activation of the PI3K-NF-kB cascade [41]. Surprisingly, while IntegroPectin treatment does not induce any significant modulation of IL-1β and IL-6 expression, we observed a significant decrease in iNOS expression, which is heavily up-regulated by NF-κB [42]. Typically, microglia cells induce iNOS expression after the exposure to inflammatory stimuli, including LPS [43]. The increase in iNOS expression leads to the sustained production of NO and the subsequent generation of reactive nitrogen oxide species (RNOS), which can mediate a broad spectrum of pathological effects, including the impairment of cell metabolism. Moreover, although NO plays several physiological roles, excessive NO generation by iNOS activation leads to the activation of inflammatory pathways and mediators, including histamine and cytokines, leading to cell damage and, ultimately, to cell death [26]. Accordingly, numerous studies document iNOS expression in a large number of human disorders [26].

In summary, although preliminary, our data suggest that the grapefruit IntegroPectin may modulate the inflammatory response and basal microglia activation by inhibiting the cascade of PI3K-NF-kB-iNOS. In support of our conclusions, a recent study concerning the in vivo administration of a similar *Citrus* pectin derived via hydrodynamic cavitation from the peels of *Citrus reticulata* (mandarin) has shown immunomodulatory activity and a reduction in LPS-induced lymphocytes in rats, though not linearly associated with dosage [44].

Among the biomolecules adsorbed at the outer surface of the grapefruit’s pectic fibers, the flavanone glycoside naringin (4′,5,7-trihydroxyflavonone-7-rhamnoglucoside) was found to be exceptionally concentrated in grapefruit IntegroPectin [14]. Due to the naringenin scavenging of free radical activity and its effects on the inhibition of the NF-κB signaling pathway and expression of inflammatory proteins, including iNOS [45], we can hypothesize a major role of this flavonone in mediating the protective effects of grapefruit IntegroPectin on microglia cells.

## 5. Conclusions

In conclusion, by studying the protective effects of grapefruit IntegroPectin on human microglial HMC3 cells we have discovered that this new *Citrus* pectin exerts a multispectral beneficial activity on microglia cells by inhibiting the intracellular pathways typically associated with apoptotic, oxidative and neuroinflammatory responses. 

Coupled with successful in vivo tests proving the powerful cardioprotective activity of this pectin [16], these results strongly support further investigations aimed at exploring the therapeutic role of this new pectin in in vivo models of oxidative stress–neuroinflammatory-based diseases, including neurodegenerative disorders. 

Featuring a particularly low (22%) degree of crystallinity and a very low degree of esterification of 14%, grapefruit IntegroPectin is uniquely rich in adsorbed naringin (74 mg/g) and in long and numerous hydrophilic RG-I regions [14]. All this favours the interaction of the IntegroPectin chains rich in hydrophilic carboxylate and hydroxyl groups with the HMC3 cell membrane, enhancing the delivery of bioactive molecules, including the otherwise poorly soluble naringin, well known for its anti-inflammatory and antioxidant activity [45]. Taking into account the recently discovered antibacterial, cardioprotective [16], mitoprotective, antioxidant and antiproliferative properties of grapefruit IntegroPectin [13], these new findings suggest that this novel pectin derived from the *Citrus paradisi* peel residues of industrial juice extraction using water and electricity constitutes one of the single most eminent examples of pectin as a universal medicine [46].

## Data Availability

The data presented in this study are available on request from the corresponding author.
